# Safe and targeted anticancer therapy for ovarian cancer using a novel class of curcumin analogs

**DOI:** 10.1186/1757-2215-6-35

**Published:** 2013-05-11

**Authors:** Kellie S Rath, Georgia A McCann, David E Cohn, Brian K Rivera, Periannan Kuppusamy, Karuppaiyah Selvendiran

**Affiliations:** 1 Division of Gynecologic Oncology, Department of Obstetrics and Gynecology, The Ohio State University, Columbus, OH 43210, USA; 2 Department of Internal Medicine, The Ohio State University, Columbus, OH 43210, USA

**Keywords:** Ovarian cancer, Targeted therapy, STAT3, Curcumin analog, Curcumin

## Abstract

A diagnosis of advanced ovarian cancer is the beginning of a long and arduous journey for a patient. Worldwide, approximately half of the individuals undergoing therapy for advanced cancer will succumb to the disease, or consequences of treatment. Well-known and widely-used chemotherapeutic agents such as cisplatin, paclitaxel, 5-fluorouracil, and doxorubicin are toxic to both cancer and non-cancerous cells, and have debilitating side effects Therefore, development of new targeted anticancer therapies that can selectively kill cancer cells while sparing the surrounding healthy tissues is essential to develop more effective therapies. We have developed a new class of synthetic curcumin analogs, diarylidenyl-piperidones (DAPs), which have higher anticancer activity and enhanced bio-absorption than curcumin. The DAP backbone structure exhibits cytotoxic (anticancer) activity, whereas the N-hydroxypyrroline (-NOH) moiety found on some variants functions as a cellular- or tissue-specific modulator (antioxidant) of cytotoxicity. The anticancer activity of the DAPs has been evaluated using a number of ovarian cancer cell lines, and the safety has been evaluated in a number of non-cancerous cell lines. Both variations of the DAP compounds showed similar levels of cell death in ovarian cancer cells, however the compounds with the -NOH modification were less toxic to non-cancerous cells. The selective cytotoxicity of the DAP–NOH compounds suggests that they will be useful as safe and effective anticancer agents. This article reviews some of the key findings of our work with the DAP compounds, and compares this to some of the targeted therapies currently used in ovarian cancer therapy.

## Introduction

“Targeted therapy” is a relatively modern term that is commonly used to describe new drugs that are specifically designed to take advantage of known molecular pathways involved in the pathophysiology to be treated. Targeted therapies include small molecules and monoclonal antibodies. A number of new small molecules and immunotherapeutic agents for cancer treatment are currently in clinical trials or in advanced development phase. This review will focus on our research efforts, specifically diarylidienyl piperidone (DAP) analogs, in the development of new targeted agents for the treatment of ovarian and other solid tumors. We will highlight the selective cytotoxicity of these agents toward cancer cells, sparing the surrounding healthy tissues. We will discuss the current challenges of ovarian cancer drug discovery, and finally identify the potential future of targeted therapy for ovarian cancer.

### New approaches for ovarian cancer therapeutics

Ovarian cancer is the leading cause of death from gynecologic cancer in North American women. In the US alone 22,280 new cases of ovarian cancer will be diagnosed and 15,500 women will die in 2013 [1]. Initial management consists of aggressive surgical cytoreduction followed by adjuvant platinum and taxane-based chemotherapy. Despite initial response in many women, 70-80% will relapse and ultimately die of their disease. Therapy at the time of relapse is less effective, and recurrent disease is uniformly fatal. Unfortunately, clinical response to second-line therapy is usually short-lived, and women with recurrent disease will die of ovarian cancer [2-4]. Finding new therapies that can reduce the rate of recurrence through overcoming resistance is essential.

With advancing technology and access to biospecimens, the ability to obtain a genetic and molecular profile of cancers has led to the understanding of cancer pathways and the development of targeted therapies [5]. The goal of these therapies is to specifically target cancer cells, while leaving normal tissues unaffected. In many solid tumors these agents have led to improvements in both progression-free and overall survival. For example, in metastatic colorectal cancer, the addition of bevacizumab to standard chemotherapy added 8 months to overall survival with a 30 month improved overall survival [6,7], unfortunately, the goal of sparing normal tissues has not been fully realized. As more patients are treated with theses targeted therapies, the adverse events associated with these agents are becoming better understood. While the traditional toxicities of cytotoxic chemotherapy are less common, adverse events such as rash, gastrointestinal toxicity (diarrhea and bowel perforation) and pulmonary toxicities are observed.

The poor prognosis associated with ovarian cancer (given that it is usually diagnosed after metastatic disease is present) makes it an optimal disease in which targeted therapies can be developed. The genetic and molecular profile of epithelial ovarian cancer (EOC) is complex, making a single molecular target difficult to identify. In EOC, overexpression of VEGF-A has been associated with advanced disease, poor prognosis, and ascites formation [8-10]. Given this, bevacizumab has been studied in both primary and recurrent settings netting an improvement of 4 months in progression free survival [11,12]. However, this did not translate to an improvement in overall survival [13]. Poly(ADP-ribose) polymerase (PARP) inhibitors were initially considered a potential treatment specifically for tumors with germline BRCA mutations due to the inherent defect in homologous recombination that occurs in BRCA-deficient tumors [14-16]. Trials are ongoing testing both of these promising targeted therapies in patients with ovarian cancer [17-19]; however, to date no targeted agent has been shown to improve overall survival in ovarian cancer nor been granted FDA approval. Table 1 reviews targeted agents of interest in ovarian cancer that have been evaluated in either Phase II or III trials. Additionally, data regarding disease site of FDA approval, response rates, and common adverse events are reported.

**Table 1:** Selected targeted agents for ovarian cancer evaluated in phase II studies*

**Targeted molecular agents**	**Primary molecular target**	**FDA approved cancer sites**	**Response rate in ovarian cancer**	**Toxicities**
**Imatinib**	KIT, PDGF-R (TKI)	GIST	0% ORR, 33% SD [20,21]	Fatigue, diarrhea, rash, nausea, cardiotoxicity, granulocytopenia
**Trastuzumab**	HER-2 (mAB)	Breast, gastroeosphageal	7.3% ORR [22]	Fatigue, diarrhea, rash, cardiotoxicity, anemia, dyspnea, neutropenia
**Pertuzumab**	HER-2 (TKI)	Breast cancer	4.3% ORR, 6.8% SD [23]	Diarrhea, neutropenia, nausea, LV dysfunction, VTE, vomiting, renal failure
**Bevacizumab**	VEGF (mAB)	Glioblastoma, NSCLC, mBreast, mCRC, mRCC	15-21% ORR, 25-52% SD [24,25]	Fatigue, diarrhea, anorexia, hypertension, gastrointestinal perforation, proteinuria, hemorrhage, congestive heart failure, arterial thromboembolism, wound healing problems
**Gefitinib**	EGFR (TKI)	NSCLC	0-4% ORR, 29-37% SD [26-28]	Diarrhea, rash, nausea, vomiting, mucositis, dyspnea
**Erlotinib**	EGFR (TKI)	NSCLC	6% ORR, 44% SD [29]	Fatigue, diarrhea, rash, anorexia
**Temsirolimus**	mTOR inhibitor	RCC	9.3% ORR, 24.1% 6m PFS [30]	Fatigue, diarrhea, rash, nausea, anorexia, stomatitis, anemia, hypertension, dyspnea
**Vandetanib**	EGFR, TEGF, RET (TKI)	Medullary thyroid cancer	0% ORR/SD [31]	Diarrhea, rash, hypertension, proteinuria, asymptomatic, QT prolongation
**Sorafenib**	VEGF, PDGF, c-Raf (TKI, Raf KI)	RCC, hepatocellular carcinoma	3% ORR, 34% SD [32]	Fatigue, diarrhea, rash, nausea, vomiting, anorexia, hypothyroidism, cardiotoxicity, hand–foot syndrome
**Sunitinib**	VEGF, PDGF, KIT (TKI)	RCC, GIST, pancreatic neuroendocrine tumor	3% ORR, 53% SD [33]	Fatigue, diarrhea, nausea, vomiting, hypothyroidism, hypertension, cardiotoxicity
**Pazopanib**	TKI VEGF, PDGFR (TKI)	RCC, soft tissue sarcoma	31% ORR, 56% SD (Ca-125 response) [34]	Fatigue, diarrhea, nausea, anorexia, hypertension, abdominal pain, arrhythmia, hepatotoxicity, hemorrhage
**Lapatinib**	HER2, EGFR (TKI)	Breast Cancer	0% ORR, 8 SD [35]	QT prolongation, CYP3A4, GI toxicity
**Olaparib**	PARP inbibitor	n/a	24-41% ORR, 35%-59% SD (BRCA carriers) [36,37]	fatigue, somnolence, nausea, loss of appetite, thrombocytopenia

*There are no FDA approved targeted agents in ovarian cancer.

**Abbreviations**: TKI = tyrosine kinase inhibitor, mAB (monoclonal antbody), mTOR (mammalian target of rapamycin), PARP (Poly ADP-ribose polymerase), GIST (Gastrointestinal stromal tumor), NSCLC (non-small cell lung cancer), CRC (colorectal cancer), RCC (renal cell carcinoma), m (metastatic), ORR (overall response rate, REIST criteria if not specified), SD (stable disease).

### Curcumin and its anti-cancer property

Curcumin (diferuloylmethane) is a major constituent of turmeric powder, a spice used extensively in Southeast Asia for centuries. This yellow pigment is extracted from the rhizomes of the plant *Curcuma longa*, and is recognized for its medicinal properties including anti-inflammatory, anti-oxidant, anti-proliferative, anti-angiogenic, and anti-tumor activities [38-43]. The anti-carcinogenic properties of curcumin have been demonstrated in animal models and human studies have shown the chemo-preventive properties of curcumin against breast, prostate, colon, and lung cancer [44-50]. Curcumin’s anti-neoplastic activity, along with its low molecular weight and apparent lack of toxicity (use of up to 8 g/day), makes it an ideal foundation for the development of new, synthetic chemotherapeutic agents [51].

### Problems with solubility and bioavailability of curcumin

Despite curcumin’s activity as an anti-cancer agent with minimal side effects, it is notorious for poor bioavailability, low solubility in aqueous solutions, and low potency [52-55]. The majority of curcumin is processed by the gut and very little is absorbed into the vascular system [56-58]. When administered orally, doses of up to 8 grams per day produce very low serum concentrations of curcumin, about 1.77 μM [59], limiting curcumin’s potential as a chemotherapeutic agent. To address this, some investigators have attempted to modify the delivery method, including the use of a nanoparticle-encapsulated form of curcumin (nanocurcumin) [60,61]. Another approach to circumvent the limitations presented by curcumin is the development of synthetic chemical analogs with enhanced solubility, bioabsorption and potency. We have published several reports on a novel class of curcumin analogs, diarylidenylpiperidones (DAPs), which have been synthesized by shortening and incorporation of a piperidone ring within the beta-diketone backbone structure of curcumin and additional fluorination of the phenyl groups [62]. In this review, we will focus on two compounds DAP-F(p) and DAP-F(p)NOH which were synthesized by our group [63].

### DAPs have superior bioavailability than curcumin

**Figure 1:**
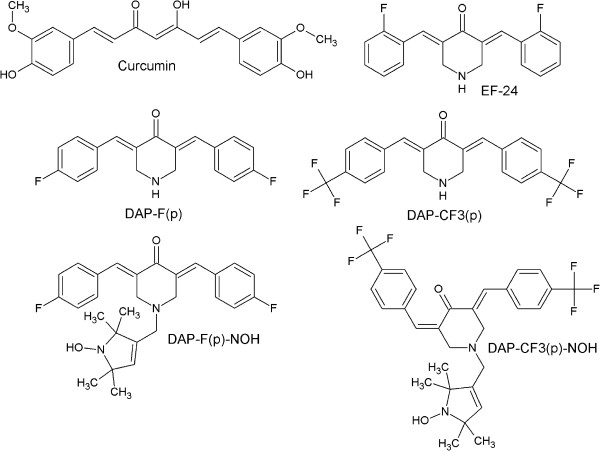
**Structures of curcumin, EF24 (3,5-bis(2-flurobenzylidene piperidin-4-one) and DAP compounds are shown.** DAP-F(p) and DAP-CF3(p) are 3,5-diarylidenyl piperidones containing *para*-fluoro substitutions on the phenyl groups. DAP-F(p)-NOH and DAP-CF3(p)-NOH contain an N-hydroxy-pyrroline moiety covalently linked to the N-terminus of the piperidone ring. (*DAP figures reproduced with permission from Free Radical Biology and Medicine/Elsevier, reference # 46*).

The DAP compounds, while structurally similar to curcumin (Figure 1), do not share the limitations of low bioabsorption and bioavailability. Two DAP compounds, DAP-F(p)-NOH and DAP-F(p), have been examined *in vitro* to determine their bioabsorption [62,64,65]. Bioabsorption of DAP-F(p)-NOH, was compared to curcumin using UV/Vis and electron paramagnetic resonance (EPR) spectrometry of cell or tissue lysates. Ovarian cancer cells grown in medium containing 10 μM of DAP-F(p)-NOH, demonstrated absorption of 220 pmol/million cells after 1 hour. In contrast, cells exposed to 100 μM of curcumin only absorbed about 20 pmol/million. Additionally, after removal of DAP-F(p)-NOH-containing culture medium and replacement with standard media, the EPR active form of DAP-F(p)-NOH was detected in cells for at least 72 hours. However, curcumin was not present in the cells at this time point. The distribution of DAP-F(p)-NOH *in vivo* was also examined using EPR spectrometry of plasma, liver, kidney and stomach tissue samples of rats exposed to the compound. These samples were collected 3 hours after intraperitoneal (IP) injection of either 10 mg/kg or 25 mg/kg of DAP-F(p)-NOH. A measurable EPR spectrum was found in liver, kidney, blood, and stomach samples, indicating the presence of DAP-F(p)-NOH in paramagnetic nitroxide form. Quantification revealed that the liver concentration of HO-3867 was twice that of other organs. Further, in a murine tumor xenograft model of ovarian cancer, animals were given DAP-F(p)-NOH in feed, and tumor tissue samples were subsequently examined with EPR. In these samples, an EPR signal was detected, indicating that oral administration of DAP-F(p)-NOH is an effective delivery method [62].

### Biological activity of DAPs

DAPs incorporate several well-established strategies to enhance metabolic stability, and increase bioavailability within the tissues [43,62,66,67]. This, in turn, translates to increased anticancer efficacy when treating cancer cells both *in vitro* and *in vivo* when compared to curcumin [65,68-70]. We investigated a number of DAP compounds using biochemical and molecular studies with the aim of identifying the connections between the structure and mode of action. Other groups have also reported upon DAP-type compounds. In 2004, Adams, et al, reported the synthesis and anticancer/antiangiogenesis screening of a number of curcumin analog compounds, including DAPs [71]. Subramaniam, et al, later focused upon one of these compounds, EF24 for an *in vivo* study, and reported high anticancer potency in colon cancer tumor xenografts [69]. Lagisetty, et al, synthesized a number of derivatives or similar compounds to EF24, and conducted structure-activity screenings [72]. One of these derivatives, CLEFMA, exhibited significantly higher cancer cell-killing potential than EF24.

### DAPs are effective against multiple human cancers

Diphenyl difluoroketone (EF24), an ortho-flourinated DAP, is toxic to a variety of cancer cells *in vitro*[64,66,73,74]. This has been attributed to cell-cycle arrest and apoptosis in both colon and ovarian cancer cell lines [66,69]. Interestingly, this toxicity was not seen when mouse embryo fibroblasts were treated with EF24, suggesting a component of selective cytotoxicity. This activity was confirmed *in vivo* with a xenograft colon cancer model, showing tumors treated with EF24 were smaller than control [69]. We have observed that DAP-F-(p), is more potent than EF24 for inducing cytotoxicity in ovarian cancer cells [70]. In our own work, we studied multiple DAP compounds, and found that they exhibit similar if not greater toxicity than cisplatin to multiple cancer cells lines in vitro. However, those compounds with the –NOH moiety were not toxic to non-cancer cell lines (Table 2). We also confirmed these findings in vivo with an ovarian cancer xeongraft model [70]. In mice treated with 100 ppm DAP-F(p)-NOH, the tumors size was reduced 70 to 80% compared to untreated. In addition the mice did not show any gross signs of toxicity measured by two profiles; body weight and feed intake [70].

**Table 2:** Growth-inhibition efficacy data on ovarian cancer cell lines and normal cell lines treated with DAP compounds compiled from individual MTT assay

**Cell lines**	**Cisplatin 10 μg/ml (%)**	**Curcumin 100 μM (%)**	**DAP-F (p) 10 μM (%)**	**DAP-F(p)-NOH 10 μM (%)**	**DAP-CF3(P) 10 μM (%)**	**DAP-CF3(P)-NOH 10 μM (%)**
**A2780**	75-85	65-75	80-90	75-85	80-90	80-90
A2780R*	50-60	55-65	85-90	70-80	75-85	70-80
**SKOV3**	80-90	65-75	80-90	75-85	85-90	75-80
**OVCAR3**	70-80	65-70	75-80	80-85	75-85	70-80
**OV-4**	75-80	60-65	75-80	70-80	80-85	80-85
**PA-1**	65-75	50	65-75	70-75	75-80	75-80
**hOSE**	☹	☺	☹	☺	☹	☺
**CHO**	☹	☺	☹	☺	☹	☺
**H9C2**	☹	☺	☹	☺	☹	☺
**HSMC**	☹	☺	☹	☺	☹	☺

Cell viability, cell survival and cell proliferation were quantified as means ± SE (N = 8, p ≤ 0.05 versus control) and expressed as percentage of respective untreated controls. ☹ = Moderately toxic; ☺ = Non-toxic; - = not tested.

### Metabolic conversion of DAPs in cells

The N-hydroxypyrroline (–NOH) moiety is capable of undergoing a reversible, one-electron oxidation to its nitroxide form, which is paramagnetic and detectable by EPR spectroscopy. Hence, we determined whether or not DAP-F(p)-NOH is converted its corresponding nitroxide form in cells. The EPR spectrum measured from a 100 μM solution of DAP-F(p)-NOH incubated with cancer cells showed a characteristic triplet feature attributable to the nitroxide form [64]. This was verified using a known nitroxide as a control. A five-fold increase in the EPR signal intensity of the nitroxide metabolite was observed in cancer cells incubated with DAP-F(p)-NOH when compared to cells treated with DMSO alone. Under these conditions, DAP-F(p), which possess the DAP backbone structures of NOH respectively, but lacks the pro-nitroxide moiety, did not show any EPR signal, suggesting that the N-hydroxypyrroline moiety is the source of the observed EPR signal. The results show the presence of a significant level of the antioxidant nitroxide form in the cells tested, and that the metabolite level was significantly higher (25–30%) in noncancerous cells when compared to cancer cells (7–16%). This difference can be explained by the intracellular environment of the cancer cells which is characterized by hypoxia, acidemia, and presence of glutathione. Cancer cells are more reducing than their normal, non-cancerous, analogues. Since HO-3867 exists in both oxidized and reduced forms, is particularly susceptible to changes in the redox state of the cellular environment. Therefore, HO-3867 undergoes loss of an electron and a greater shift from the antioxidant to anti-proliferative form in cancer cells.

### Superoxide radical-scavenging activity of DAPs

Many chemotherapeutic agents act by producing free radicals, which increases the oxidative stress [75,76]. N-hydroxypyrroline-bearing compounds and other nitroxides are generally known to have antioxidant properties including superoxide dismutase- and catalase-mimetic activities [77]. The superoxide radical scavenging ability of DAPs was evaluated using a competitive reaction in the presence of DEPMPO (5-(diethoxyphosphoryl)-5-methyl-1-pyrroline-N-oxide), an EPR spin-trap commonly used for this assay [78,79]. Superoxide radicals were generated using an aerobic solution of xanthine and xanthine oxidase (X/XO) and detected as DEPMPO–OOH adduct by EPR spectroscopy. The DAP compounds (100 μM) were used to compete with 1 mM DEPMPO for the superoxide ions. SOD (4.2 μM) was used as a positive control. The EPR studies clearly demonstrated that the N-hydroxypyrroline modified DAPs are capable of scavenging superoxide radicals.

### Selective induction of ROS by DAP-F(p)-NOH

Several studies have shown that curcumin and curcumin analogs induce apoptosis and inhibit growth in human cancer cells [66,73,80-82]. Curcumin is also known for its antioxidant properties and ability to act as a free-radical scavenger by inhibiting lipid peroxidation and oxidative DNA damage [83]. Several *in vitro* studies, however, suggest that curcumin analog-induced apoptosis is associated with ROS production and/or oxidative stress in cells [64,73,84,85]. Curcumin also has demonstrated anticancer activity by decreased mitochondrial membrane potential and induced reactive oxygen species (ROS) in pancreatic cancer cells [86]. Apoptosis is induced through an increase in intracellular ROS concentration through reduction of the mitochondrial membrane potential (MMP), alterations in MAPK (Mitogen-activated protein kinases) expression, and activation of JNK (Jun-amino-terminal kinase), p38 and ERK (Extracellular signal-regulated kinases) [87-89]. GSH (Glutathione) and NAC (N-acetylcysteine), both antioxidants, have been shown to block curcumin-induced ROS production and MMP loss, and rescue cells from curcumin-induced apoptosis. This suggests that curcumin induces apoptosis in cancer cells through a ROS-dependent mitochondrial signaling pathway, and alterations in signaling pathways. In addition, it has been reported that curcumin is a weak stimulator of differentiation and shows synergistic effects on retinoid acid induced differentiation of cancer cells [89]. The production of ROS has been linked to the anti-proliferative effects of most agents [90-92].

We further determined whether the DAPs could have a similar effect upon cancer cells. Cancer cells were incubated with 5 or 10 μM of the DAP compounds DAP-F(p)-NOH, and DAP-F(p) for 12 h, and intracellular ROS generation was measured by DCF (dichloroflurescein) fluorescence [64]. The fluorescence intensity observed in cancer cells was significantly higher in the cells treated with both DAP compounds when compared to untreated controls. The DCF fluorescence intensity in human smooth muscle cells (HSMCs) treated with DAP-F(p)-NOH was not significantly different from that of the untreated cells. In contrast, DAP-F(p) induced significant ROS generation in HSMC cells. The results show that DAP-F(p) and DAP-F(p)-NOH are comparable in inducing ROS generation in cancer cells. However, in normal cells (HSMCs), DAP-F(p)-NOH generated significantly less ROS when compared to DAP-F(p) [64]. Taken together, the results imply that DAP-F(p)-NOH is capable of inducing oxidative stress in cancer cells while sparing healthy cells. These cells are spared because in the normal cellular environment, unlike the cancer cellular environment, the equilibrium shift to loss of an electron and an increase in the anti-oxidant form of the compound.

### Efficacy in treating *in vivo* ovarian carcinoma xenografts

The anti-cancer efficacy of curcumin was first confirmed in a model of Dalton's lymphoma cells grown as ascites, and curcumin was found to reduce the development of animal tumors [93]. Curcumin has been shown to prevent cancer of the skin, colon, stomach, liver, lung, and breast through oral administration using *in vivo* rodent models of these cancers [94-97]. The effects of dietary curcumin on colon carcinogenesis, in particular, have been demonstrated in both chemically-induced and genetically-modified animal models [98]. Furthermore, curcumin has been shown to be a chemopreventive agent by inhibiting tumorigenesis during the initiation phase in chemical models [99]. Based on our *in vitro* results, which showed significant cytotoxicity of DAPs to human various cancer cell lines, we evaluated the efficacy of DAPs in a human ovarian tumor xenograft grown on the back of immunocompromised mice. A significant reduction in the tumor volume was observed in a dose-dependent manner [70]. Doses of 50 and 100 parts-per-million (ppm) were effective in reducing tumor growth when compared to vehicle-treated controls. Tumor weight in untreated animals measured 1.2g compared to 0.6g and 0.2g in the 50 and 100 ppm groups respectively. This translates to a 70 to 80% reduction of tumor growth observed in the group treated with 100 ppm of HO-3867. Animals treated through oral administration of DAP-F(p)-NOH did not show any gross signs of toxicity and/or possible adverse side effects as measured by two parameters: body weight and diet consumption. Many cancer patients exposed to modern chemotherapeutics experience the undesirable side effects of a loss of appetite and subsequent weight loss, often extreme. Our results demonstrated *in vivo* antitumor efficacy using DAP-F(p)-NOH without any toxicity complications. The results of our *in vitro* and *in vivo* studies using DAP-F(p)-NOH imply that the induction of apoptosis may be an additional means by which DAP-F(p)-NOH inhibits ovarian tumor growth.

## Understanding the molecular targets of DAPS in ovarian cancer

Recent evidence suggests that curcumin analogs have a wide range of molecular targets, which supports the notion that curcumin analogs influence numerous biological and molecular cascades [45,100]. Included among the DAP molecular targets are transcription factors, growth factors and their receptors, and genes regulating cell proliferation and apoptosis. We have shown that the DAP compounds target the signal transducer and activator of transcription 3 (STAT3) signaling pathways in various cancer cells when compared to other pro-oncogenic signaling pathways [64,70].

### Induction of cell-cycle arrest by activation of p53

Cell-cycle control plays a critical role in the regulation of tumor cell proliferation. Cell-cycle is tightly controlled in normal cells by checkpoints and these checkpoints can become disrupted by damaged DNA [101]. The cell-cycle consists of four phases (G1, S, G2 and M). p53 is a nuclear transcription factor that accumulates in response to cell-cycle arrest and DNA damage [102]. This triggers transcriptional trans-activation of p53 target genes such as p21, and p27, leading to cell-cycle arrest, or apoptosis [103]. Another p53 transcriptional target is the Mdm2 gene, whose protein product ubiquitinates p53 and targets it for proteasome-mediated degradation [102].

The failure of many chemotherapeutic agents reflects an inability of these drugs to induce cell- cycle arrest and apoptosis [104]. The cell-cycle and programmed cell death are intimately related, as evidenced by the central role of p53 in both cell-cycle arrest and in the induction of apoptosis. Many cytotoxic agents arrest the cell cycle at the G1, S, or G2-M phase [105-107]. DAPs induced G1/G2-M cell cycle arrest in cisplatin-resistant ovarian cancer cells and serum-stimulated vascular smooth muscle cells [66,67]. Previous studies have shown that the G2-M-phase progression is regulated by a number of Cdk/cyclins as well as Cdk inhibitors such as p21 and p27. We have observed that the curcumin analog-induced G2-M cell-cycle arrest is mediated by the induction of p53 and p21 and downregulation of cyclin A and Cdk2 [108]. Previous studies also have shown that the synthetic curcumin analog, EF24, induces G2/M cell-cycle arrest by means of a redox-dependent mechanism in human breast cancer cells and human prostate cancer cells [73].

### Induction of apoptosis

The induction of apoptosis is believed to be the main mechanism of action for the anticancer effects of flavonoids, although other mechanisms have been proposed [109,110]. Some of these alternatives include cell-cycle inhibition by inactivation of cyclin-dependent kinase 2 (CDK-2), and attenuation of angiogenesis by inhibition of vascular endothelial growth factor (VEGF)–induced phosphatidylinositol-3-kinase (PI3K) activity [111]. Many curcumin derivatives induce apoptosis in cancer cells, but the mechanisms by which they do so differ [66,70,112-114]. The death receptor–associated mechanism has been recently receiving much attention for the anticancer activity of curcumin derivatives [82,115]. We reported that the death receptor gene Fas/CD95 and FASL were activated in cancer cells by curcumin analogs [66,70]. We further observed that the expression level of TNF-R1, the receptor of tumor necrosis factor-α, was unchanged in DAPs–treated cancer cells. It has been reported that curcumin promoted tumor necrosis factor-α–induced apoptosis in a variety of cancer cells, but without a significant increase in the TNF-R1 expression level. Curcumin and curcumin analogues have also been shown to upregulate death receptor 5 and FasL expression, thereby inducing apoptosis in human cancer cells [82,115,116]. Upregulation of the death receptor superfamily–mediated signaling appear to be critical involvement in the stimulation of apoptosis following curcumin analog exposure.

Several reports have shown that some anticancer agents induced apoptosis, in part, by blocking the activation of Akt [117,118]. Akt prevents cells from undergoing apoptosis by inhibiting pro-apoptotic factors and suppressing death-receptor signals such as Fas and FasL [66]. The role of Akt signaling in regulating death receptor signaling is not fully understood. In a recent study using prostate cancer cells and T-lymphocytes, blocking Akt-signaling has been shown to increase caspase-8 activity, resulting ultimately in FasL-dependent apoptosis [119]. It reported that the curcumin analogs also downregulate Akt signaling and induces apoptosis [66,120]. Akt activation appears to be a critical downstream target of the death receptor-mediated apoptotic pathway, and should provide opportunities as a target for future anticancer therapeutics.

### Targeting STAT3 and PTEN

Signal transducer and activator of transcription 3 (STAT3) has been implicated in the pathogenesis of a variety of human malignancies, including head and neck cancer, myeloma, prostate cancer, breast cancer, colon cancer, and ovarian cancer [121]. Activation of STAT3 can be accomplished by the Janus kinases (JAKs; including tyrosine kinase 2, TYK2), activated epidermal growth factor receptor (EGFR), and Src kinase [122-124]. STAT3 is constitutively activated in many tumor types and this activation promotes acceleration of cell proliferation, upregulation of survival factors, and activation of anti-apoptotic proteins [125]. Activated STAT3 imparts cellular resistance to chemotherapy by inhibiting apoptosis in epithelial malignancies, including ovarian cancer [126-128]. Currently, the oncogenic transcription factor STAT3 has attracted much attention as a pharmacologic target, although *in vivo* evidence demonstrating that inhibition of STAT3 could counteract cancer remains incomplete [129].

Curcumin and curcumin analogs induce apoptosis by inhibiting pSTAT3 Tyr^705^ and Ser^727^ expression in various cancer cells [65]. Flavonoids and other analogs have been shown to target STAT3 indirectly, by inhibiting STAT3-regulating genes such as the JAK1 and JAK2 pathways [64,81,130]. We have reported that the DAPs caused a substantial inhibition of phospho-JAK1 (Tyr^1022/1023^), suggesting that DAPs can inhibit the constitutive activation of STAT3, which may be caused, at least in part, by the inhibition of pJAK1 [70]. Further, evidence showed that DAPs activates cleaved caspase-3 and induces apoptotic markers of PARP in various cancer cell lines, suggesting that DAPs induce apoptosis in cancer cells by targeting STAT3 proteins [64]. Many reports have shown that blockage of constitutive STAT3 activation and signaling results in growth inhibition and induction of apoptosis in tumor cells both *in vitro* and *in vivo*[131-133]. However, DAP-F(p)-NOH may also inhibit STAT3 activation through JAK2, Src, Erb2, and epidermal growth factor receptor (EGFR), which are implicated in STAT3 activation as well. Cisplatin resistance is associated with the altered activation of signaling pathways which include phosphatidylinositol 3-kinase PI3K/Akt and MAPK, or the suppression of tumor suppressor genes, p53 and PTEN [134,135]. The tumor suppressor gene PTEN encodes a multifunctional phosphatase that is mutated in a variety of human cancers [136,137]. PTEN is considered to be a central regulator of cell proliferation and apoptosis [138]; inactivation of PTEN results in increased Akt activity in many cancers [135,139,140]. The overexpression of PTEN in cancer cells carrying mutant or deletion-type PTEN can inhibit cell proliferation and tumorigenicity via induction of cell cycle arrest at the G1 phase and apoptosis.

Previously, we have reported that the curcumin analog EF24 downregulated pAkt Ser^473^ and Thr^308^ through the upregulation of PTEN expression in cisplatin-resistant (CR) ovarian cancer cells [66]. Overexpression of PTEN showed inhibition of Akt activation, further supporting the idea that PTEN upregulates p53 through the Akt pathway, and how it might be involved in cell- cycle arrest and apoptosis in CR ovarian cancer cells. Further we showed that EF24 might inhibit PTEN proteasomal degradation, leading to the accumulation of polyubiquitinated proteins in the cells. These results suggest that EF24 may exert its cytotoxic effect on cancer cells through inhibition of the pPTEN and PTEN proteasome degradation [66]. This is consistent with previous studies, which showed that the turnover of PTEN in cultured COS-7 cells was dependent mainly on proteasomal degradation [141]. The oncogenic potential of PTEN is further highlighted by its roles in integrin signaling and ability to dephosphorylate FAK that can reduce cell adhesion and enhance migration [142]. Curcumin analogs which initiate PTEN stabilization may play a role in the regulation of cell proliferation in cancer and smooth muscle cells.

### Effects on migration/invasion and FAS/FAK pathways

Tumor progression involves complex processes that include malignant transformation, proliferation, invasion, and metastasis of cancer cells. Particularly, cancer-cell invasion and metastasis are the critical processes that define the aggressive phenotype of human cancers and pose major impediments to treatment [143,144]. Tumor cell migration requires the concerted effort of a number of molecules such as integrins, ion channels, cell adhesion molecules, soluble cytokines and growth factors, matrix-degrading proteases, and Rho GTPases [143]. The migration process involves the assembly and disassembly of focal adhesions. This process is stimulated extracellularly and is initiated by integrins and intracellular signaling proteins located in focal adhesions [145]. Focal adhesion kinase (FAK), a tyrosine receptor kinase, is activated in focal adhesions and is important in cell–extracellular matrix (ECM) interactions that affect cell migration, proliferation, and survival [145]. DAPs effectively inhibit ovarian cancer cell migration and invasion by altering the FASN and FAK expression. Further, it also exhibits the potential to inhibit vascular endothelial growth factor (VEGF)-induced invasion and migration of these cell lines [65].

FASN is overexpressed in ovarian cancer, and overexpression has been related with aggressive biologic behavior, suggesting a functional role for fatty acid synthesis in the growth, survival, and expansion/proliferation of cancer cells [146]. Recent evidence shows that FASN inhibition induces apoptosis, via inactivation of pAKT and dephosphorylation of Bad in human cancer cells, including ovarian cancer cells [147]. DAP-F(p)-NOH targets FASN, resulting in inhibition of migration and invasion in ovarian cancer cell lines [65]. A recent report also showed that suppression of growth and invasiveness of renal cancer cells by targeting FASN using a synthetic pharmacological inhibitor [148].

## Conclusion & future direction

**Figure 2:**
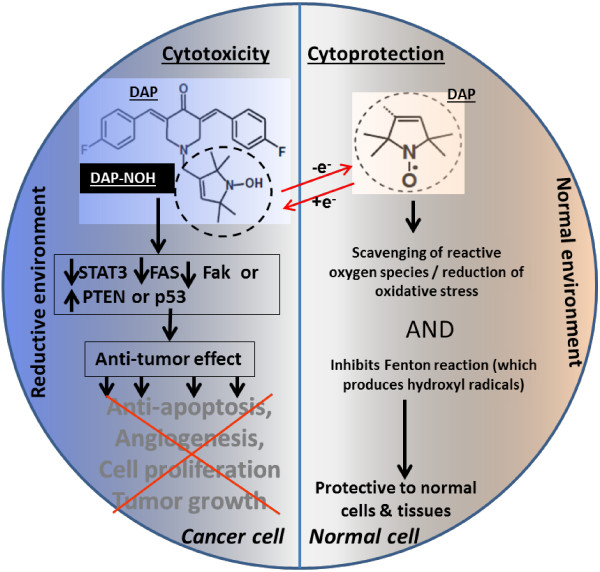
**The dual functionality of DAP-F(p)-NOH or DAP-CF3(p)-NOH compounds.** The reductive environment of the cancer cells shifts the equilibrium balance, such that less antioxidant cytoprotection is afforded to cancer cells versus normal cells. The –NOH (N-hydroxy-pyrroline) moiety undergoes conversion to and exists in equilibrium with the nitroxide (>NO) form (shown in the circle).

The diagnosis of an advanced cancer, such as ovarian, means multi-modality therapy for most patients, which typically includes chemotherapy. Unfortunately, most chemotherapy is toxic not only to tumors, but also to healthy tissue. This adversely impacts quality of life both during and after therapy. In addition, most ovarian cancer patients will ultimately recur, and die of their disease. Current cancer research focuses on developing new therapies to both improve survival and decrease toxicity. Given the anti-cancer properties of curcumin we have focused on developing compounds based that are not restricted by poor bioavailability, limited solubility, and low potency. One of these compounds DAP-F(p)-NOH shows promise not only in its toxicity toward cancer cells through a variety of mechanisms, but also its protection of healthy tissue. We show that the –NOH moiety provides an antioxidant protection to healthy cells minimizing damage to normal tissues in vivo. The cytotoxicity is mediated through the STAT3 and FAS/FAK signaling pathways (Figure 2).

While much has been learned from the collective work involving curcumin derivatives and DAP compounds in particular, it is clear that many questions are left unanswered. In the future, it will be critical to expand the existing knowledge of the mechanism of action of DAP compounds; in particular the other cancer-promoting pathways on which it may act. Additional *in silico* studies may be conducted to optimize the structure of the DAP compounds with the intent of maximizing interaction with known and future molecular targets. Further in vivo work is needed with head-to-head comparisons of DAP compounds against both standard chemotherapies and targeted agents as progress is made toward possible clinical trials. Because of the complexity and variety of cancers found in the modern world, the idea of a “silver bullet” treatment or cure is unlikely to be found. However, the continued development of new therapies such as those described here will lead to improved survival and quality of life for cancer patients.

## Competing interests

The authors declare that they have no competing interests.

## Authors’ contributions

This is a review article of previously-published work. KR, GM, DC, BR, PK, and KS all contributed to the writing, proofreading and editing of this review. All authors have read and approved the final manuscript.
